# A retrospective comparative study of a full-course precision MDT nursing studio model integrating rational emotive behavior therapy for standardized health management during pregnancy

**DOI:** 10.1097/MD.0000000000050992

**Published:** 2026-10-23

**Authors:** Li Zhang, Hongying Tao, Lingna Zhu, Shoufu Liu, Yanhui Yang

**Affiliations:** a Obstetrics Department, West China Hospital of Sichuan University-Ziyang Hospital · Ziyang Central Hospital, Ziyang, Sichuan, China.

**Keywords:** acceptability, antenatal care, feasibility, health management, multidisciplinary team, pregnancy, rational emotive behavior therapy

## Abstract

This study evaluates the clinical application of a full-course precision multidisciplinary team (MDT) nursing studio model integrating rational emotive behavior therapy (REBT) during standardized health management in pregnancy and explores its associations with maternal psychological status, antenatal care adherence, implementation-related indicators, and maternal and neonatal outcomes. This single-center retrospective comparative study included 210 pregnant women who received standardized antenatal health management from June 2024 to December 2025. Participants were classified into a conventional care group (n = 102) and an intervention group (n = 108) according to the nursing management model documented in routine clinical records. The intervention group received the MDT-REBT model in addition to routine care. Outcomes included maternal psychological indicators, health management performance, implementation- and acceptability-related indicators, and maternal and neonatal outcomes. Compared with the conventional care group, the intervention group had significantly lower generalized anxiety disorder-7, Patient Health Questionnaire-9, fear of childbirth, and perceived stress scores, as well as lower rates of moderate-to-severe anxiety and depression (all *P* < .01). Adherence to antenatal visits, gestational weight management, health education compliance, exercise compliance, dietary management compliance, sleep management compliance, self-monitoring compliance, and mean number of antenatal visits were all significantly better in the intervention group (all *P* < .05). Follow-up completion, attendance at nutritional counseling and psychological support sessions, stage-specific follow-up attendance, management compliance, and satisfaction were also significantly higher, whereas withdrawal/noncompletion was lower (all *P* < .01). Multivariable analyses showed that the intervention model was independently associated with better antenatal care adherence, improved gestational weight management, and lower generalized anxiety disorder-7 scores. Several maternal and neonatal outcomes showed numerical between-group differences, but most were not statistically significant. The full-course precision MDT nursing studio model integrating REBT was associated with improved maternal psychological status, better antenatal care adherence, enhanced pregnancy health management performance, and more favorable implementation-related indicators during pregnancy. These observational findings do not establish causality, and no clinical effectiveness conclusion can be drawn for maternal or neonatal endpoints that were not statistically significant. Further prospective multicenter studies are needed to confirm these findings.

## 1. Introduction

Pregnancy is a critical stage in a woman’s life and is accompanied by substantial physiological, psychological, and social changes. During this period, pregnant women are required not only to adapt to hormonal fluctuations and physical discomfort, but also to cope with concerns about fetal development, childbirth, role transition within the family, and future parenting responsibilities. Under the combined influence of these factors, many women experience varying degrees of emotional distress, including anxiety, depressive symptoms, excessive worry, and fear of childbirth.^[1]^ These psychological challenges may adversely affect antenatal care adherence, self-management behaviors, and maternal well-being, and may also have implications for offspring development and perinatal health outcomes.^[2–4]^

Standardized health management during pregnancy is essential for promoting maternal and neonatal health. The World Health Organization has emphasized that antenatal care should not only focus on reducing morbidity and mortality, but should also support a positive pregnancy experience through person-centered, effective, and continuous care.^[5]^ However, in routine clinical practice, antenatal nursing often remains centered on obstetric examinations, general health education, and basic follow-up. Although these components are necessary, they may be insufficient to address the complex and changing needs of pregnant women, particularly those with greater psychological burden or poorer health-related behaviors.^[6]^ As a result, some women continue to demonstrate poor compliance with scheduled antenatal visits, suboptimal lifestyle management, and limited capacity to cope with pregnancy-related stress.

In recent years, multidisciplinary team (MDT)-based care has received increasing attention in maternal healthcare. By involving obstetricians, specialist nurses, dietitians, psychological counselors, and other relevant professionals, MDT-based models may facilitate more comprehensive assessment, coordinated intervention, and individualized support across the course of pregnancy. Such an approach is particularly relevant in the context of standardized pregnancy health management, which requires not only medical monitoring and risk assessment, but also behavioral guidance, psychosocial support, and sustained follow-up.^[7]^ At the same time, growing attention has been given to the importance of perinatal mental health within routine maternity care, and contemporary evidence suggests that emotional problems during pregnancy are common and clinically meaningful.^[8]^

Rational emotive behavior therapy (REBT), as a cognitive-behavioral approach, may offer additional value in pregnancy care. REBT focuses on identifying and modifying irrational beliefs that contribute to maladaptive emotional and behavioral responses. During pregnancy, these beliefs may manifest as catastrophic interpretations of physical symptoms, excessive fear regarding fetal safety, or unrealistic expectations concerning childbirth and motherhood. Such cognitions may aggravate emotional distress and reduce engagement in health management. Evidence from systematic reviews and meta-analyses indicates that antenatal psychological interventions, including cognitive-behavioral and mindfulness-based approaches, may improve anxiety, depression, stress, fear of childbirth, and coping during the perinatal period.^[9]^ Collaborative psychological interventions have also shown benefit in reducing anxiety among pregnant women, further supporting the value of integrating structured psychological support into routine care.^[10]^

Based on these considerations, a full-course precision MDT nursing studio model integrating REBT has gradually been introduced into routine clinical practice in our institution. This model combines multidisciplinary collaboration, risk stratification, individualized management planning, cognitive-emotional intervention, and continuous follow-up throughout pregnancy. Its purpose is to provide more targeted and standardized support for pregnant women while also addressing their psychological and behavioral needs. However, evidence regarding the clinical application of this integrated model in real-world settings remains limited, particularly with respect to maternal psychological status, pregnancy health management performance, and maternal and neonatal outcomes.^[10]^ Therefore, this retrospective study aimed to evaluate the clinical application of a full-course precision MDT nursing studio model integrating REBT for standardized health management during pregnancy, and to analyze its associations with maternal psychological status, antenatal care adherence, pregnancy health management indicators, and maternal and neonatal outcomes.

## 2. Methods

### 2.1. Study design and setting

This study was approved by the Ethics Committee of West China Hospital of Sichuan University-Ziyang Hospital · Ziyang Central Hospital. This was a single-center retrospective comparative study conducted in the Department of Obstetrics. Clinical and nursing management records of pregnant women who received standardized antenatal health management between June 2024 and December 2025 were reviewed. According to the nursing management model documented in the medical records, eligible participants were classified into a conventional care group and an intervention group. Group allocation was not determined by a research randomization procedure. Because this was a retrospective study, the available analytic records identified the care model actually received but did not contain a standardized variable documenting the operational reason why an individual woman received 1 model rather than the other. Therefore, the possible influence of routine-service factors on care-model selection could not be reliably reconstructed, and potential selection bias should be considered when interpreting the between-group comparisons.

### 2.2. Participants

Pregnant women were eligible for inclusion if they met all of the following criteria: singleton pregnancy, establishment of antenatal care records at the study hospital, receipt of standardized pregnancy health management during the study period, and availability of complete demographic, obstetric, psychological, and follow-up data in the hospital information system.The exclusion criteria were preexisting severe psychiatric disorders, severe medical or obstetric diseases requiring prolonged inpatient management, transfer to another institution before delivery, and missing key data required for primary outcome assessment.

### 2.3. Grouping

Participants were divided into 2 groups based on the nursing management model documented as having been received during pregnancy. The conventional care group received routine antenatal nursing care. The intervention group received a full-course precision MDT nursing studio model integrating REBT in addition to routine care. The final analysis included 102 women in the conventional care group and 108 women in the intervention group. No research-specific allocation algorithm was applied retrospectively; group membership reflected routine clinical care as recorded in the nursing documentation.

### 2.4. Nursing management models

Women in the conventional care group received routine antenatal nursing management, including regular obstetric examinations, general prenatal education, routine nutritional and lifestyle guidance, and standard follow-up according to departmental practice.Women in the intervention group received, in addition to routine care, a full-course precision MDT nursing studio model integrating REBT. The MDT consisted of obstetricians, specialist obstetric nurses, psychological counselors, dietitians, and other relevant healthcare professionals according to clinical needs. The intervention mainly included baseline assessment and risk stratification, individualized health management planning, REBT-based psychological support, multidisciplinary follow-up, and dynamic management adjustment across different stages of pregnancy.Baseline assessment and risk stratification covered demographic characteristics, obstetric history, prepregnancy body mass index (BMI), family support, psychological status, and pregnancy-related risk factors. Individualized health management planning included stage-specific recommendations for antenatal visits, diet, physical activity, sleep, self-monitoring, and blood glucose management when indicated. REBT-based psychological support focused on identifying pregnancy-related irrational beliefs, emotional distress, fear of childbirth, and maladaptive coping patterns, followed by cognitive restructuring and supportive counseling. Multidisciplinary follow-up included nutritional counseling, psychological support sessions, and stage-specific follow-up throughout pregnancy. Dynamic management adjustment was conducted according to maternal condition, compliance, and follow-up findings.Because the intervention was delivered as a multicomponent care package, the present study was designed to evaluate the overall integrated model rather than the independent effect of REBT or any single component.

### 2.5. Data collection

Data were retrospectively extracted from the hospital electronic medical record system, antenatal nursing records, and follow-up documentation by trained investigators using a standardized case report form. Extracted variables included demographic and socioeconomic characteristics, including age, education level, employment status, household income, marital status, smoking exposure during pregnancy, alcohol consumption during pregnancy, and family support score. Obstetric and clinical variables included gestational week at enrollment, gravidity, parity, primipara status, history of miscarriage or abortion, history of cesarean section, planned pregnancy, prepregnancy weight, height, prepregnancy BMI, advanced maternal age, prepregnancy overweight or obesity, history of infertility treatment, history of gestational diabetes, history of hypertensive disorders of pregnancy, family history of diabetes, family history of hypertension, and baseline obstetric risk factors. Psychological variables included baseline and follow-up generalized anxiety disorder (GAD)-7 and Patient Health Questionnaire (PHQ)-9 scores, fear of childbirth score, and perceived stress score. Baseline GAD-7 and PHQ-9 scores corresponded to the initial antenatal management assessment. Follow-up psychological outcomes were extracted from subsequent antenatal follow-up documentation. The retrospective dataset used for this analysis did not contain a uniform gestational-week variable linked to every follow-up psychological assessment; therefore, an identical assessment week across all participants and between groups could not be confirmed. Pregnancy health management variables included adherence to scheduled antenatal visits, gestational weight management, health education compliance, exercise compliance, dietary management compliance, sleep management compliance, self-monitoring compliance, blood glucose control in women requiring glucose management, and mean number of antenatal visits. Implementation- and acceptability-related variables included follow-up completion, attendance at nutritional counseling, attendance at psychological support sessions, attendance at stage-specific follow-up, management compliance, satisfaction score, high satisfaction rate, and withdrawal or noncompletion. Maternal and neonatal outcomes included gestational diabetes mellitus, hypertensive disorders of pregnancy, cesarean section, preterm birth, postpartum hemorrhage, premature rupture of membranes, gestational anemia, operative vaginal delivery, birth weight, macrosomia, low birth weight, neonatal intensive care unit (NICU) admission, Apgar scores, neonatal jaundice, and fetal distress.

### 2.6. Outcome measures

Psychological outcomes included the GAD-7 score, PHQ-9 score, fear of childbirth score, perceived stress score, and the proportions of women with moderate-to-severe anxiety and moderate-to-severe depression. Baseline GAD-7 and PHQ-9 scores were also collected for assessment of group comparability. Follow-up psychological scores were taken from routine antenatal follow-up records. Because a uniform gestational week for these assessments was not available in the analytic dataset, variation in assessment timing could not be excluded. Pregnancy health management outcomes included adherence to scheduled antenatal visits, appropriate gestational weight management, compliance with health education, exercise compliance, dietary management compliance, sleep management compliance, self-monitoring compliance, satisfactory blood glucose control among women requiring glucose management, and the mean number of antenatal visits. Implementation-related and acceptability-related indicators included follow-up completion rate, attendance at nutritional counseling, attendance at psychological support sessions, attendance at stage-specific follow-up, management compliance, satisfaction score, proportion with high satisfaction, and withdrawal or noncompletion. These indicators were derived from documented nursing follow-up and service records. Maternal outcomes included gestational diabetes mellitus, hypertensive disorders of pregnancy, cesarean section, preterm birth, postpartum hemorrhage, premature rupture of membranes, gestational anemia, and operative vaginal delivery. Neonatal outcomes included birth weight, macrosomia, low birth weight, NICU admission, 1-minute and 5-minute Apgar scores, neonatal jaundice, and fetal distress. Fear of childbirth score, family support score, satisfaction score, and several management-related indicators were derived from routine institutional assessment tools used in the nursing management process. These measures were applied for clinical management purposes and were not all based on formally validated research instruments.

### 2.7. Definitions

For the management-related binary indicators, classification was based on the status recorded in the department’s routine nursing management and follow-up forms rather than on research-specific cutoffs created for this study. Appropriate gestational weight management, exercise compliance, dietary management compliance, sleep management compliance, self-monitoring compliance, health education compliance, and overall management compliance were coded as compliant/appropriate when the corresponding routine record indicated that the patient met the department’s individualized or stage-specific management target, and as noncompliant/not appropriate when the record indicated that the target was not met.

Advanced maternal age was defined as maternal age ≥ 35 years. Prepregnancy BMI was calculated as weight in kilograms divided by height in meters squared. BMI categories were defined as underweight (< 18.5 kg/m^2^), normal weight (18.5–23.9 kg/m^2^), and overweight or obesity (≥ 24.0 kg/m^2^). High satisfaction was defined as a satisfaction score ≥ 90. Moderate-to-severe anxiety was defined as a GAD-7 score ≥ 10, and moderate-to-severe depression was defined as a PHQ-9 score ≥ 10. Satisfactory blood glucose control was assessed only among participants requiring glucose management during pregnancy and was defined according to the departmental glycemic management protocol.

### 2.8. Statistical analysis

Statistical analyses were performed using IBM Statistical Package for the Social Sciences Statistics for Windows, version 26.0 (IBM Corp). Continuous variables were tested for normality before analysis. Normally distributed continuous variables were expressed as mean ± standard deviation and compared between groups using the independent-samples *tt*-test. Continuous variables not meeting normality assumptions were expressed as median (interquartile range) and compared using the Mann–Whitney *U* test. Categorical variables were presented as number (percentage) and compared using the chi-square test or Fisher exact test, as appropriate. To further evaluate factors associated with key study outcomes, multivariable regression analyses were performed. Binary outcomes, including adherence to antenatal visits and appropriate gestational weight management, were analyzed using multivariable logistic regression, and results were reported as odds ratio (OR)s with 95% confidence interval (CI)s. Continuous psychological outcomes, including GAD-7 score, were analyzed using multivariable linear regression, and results were reported as regression coefficients with 95% CIs. Covariates included maternal age, prepregnancy BMI, primipara status or parity, and baseline obstetric risk factors. A 2-sided *P* value < .05 was considered statistically significant.

## 3. Results

### 3.1. Baseline characteristics

A total of 210 pregnant women were included in the final analysis, including 102 in the conventional care group and 108 in the intervention group. The mean age was 29.84 ± 4.12 years in the conventional care group and 30.17 ± 4.05 years in the intervention group. The mean gestational week at enrollment was 11.26 ± 2.48 weeks and 11.51 ± 2.37 weeks, respectively. No significant differences were observed between the 2 groups in age, gestational week at enrollment, height, prepregnancy weight, prepregnancy BMI, gravidity, parity, primipara status, history of miscarriage or abortion, history of cesarean section, planned pregnancy, marital status, education level, employment status, monthly household income, smoking exposure during pregnancy, alcohol consumption during pregnancy, family support score, baseline obstetric risk factors, family history of diabetes or hypertension, baseline GAD-7 score, baseline PHQ-9 score, or baseline health management compliance score (all *P* > .05). No statistically significant baseline differences were detected for these measured characteristics (all *P* > .05); however, the observational design does not exclude unmeasured differences between groups (Table 1)

**Table 1:** Baseline demographic, obstetric, and clinical characteristics of the participants.

Variable	Conventional care group (n = 102)	Intervention group (n = 108)	Statistic	*P* value
Age, yrs	29.84 ± 4.12	30.17 ± 4.05	*t* = 0.59	.554
< 30 yrs, n (%)	56 (54.9)	55 (50.9)	*χ*^2^ = 0.34	.559
≥ 30 yrs, n (%)	46 (45.1)	53 (49.1)		
Gestational week at enrollment	11.26 ± 2.48	11.51 ± 2.37	*t* = 0.75	.454
First trimester enrollment, n (%)	81 (79.4)	89 (82.4)	*χ*^2^ = 0.31	.575
Second trimester enrollment, n (%)	21 (20.6)	19 (17.6)		
Height, cm	160.84 ± 4.91	161.22 ± 5.03	*t* = 0.55	.584
Prepregnancy weight, kg	57.96 ± 8.47	57.38 ± 7.95	*t* = 0.51	.609
Prepregnancy BMI, kg/m^2^	22.41 ± 3.27	22.08 ± 3.11	*t* = 0.75	.452
Underweight (< 18.5), n (%)	10 (9.8)	12 (11.1)	*χ*^2^ = 0.43	.807
Normal weight (18.5–23.9), n (%)	63 (61.8)	70 (64.8)		
Overweight/obesity (≥ 24.0), n (%)	29 (28.4)	26 (24.1)		
Gravidity	1.93 ± 0.88	1.87 ± 0.81	Z = 0.52	.606
1, n (%)	45 (44.1)	50 (46.3)	*χ*^2^ = 0.18	.914
2, n (%)	31 (30.4)	34 (31.5)		
≥ 3, n (%)	26 (25.5)	24 (22.2)		
Parity	0.61 ± 0.57	0.58 ± 0.54	Z = 0.39	.697
Primipara, n (%)	58 (56.9)	66 (61.1)	*χ*^2^ = 0.38	.540
Multipara, n (%)	44 (43.1)	42 (38.9)		
History of miscarriage/abortion, n (%)	27 (26.5)	24 (22.2)	*χ*^2^ = 0.52	.469
History of cesarean section, n (%)	9 (8.8)	8 (7.4)	*χ*^2^ = 0.14	.712
Planned pregnancy, n (%)	73 (71.6)	80 (74.1)	*χ*^2^ = 0.17	.678
Marital status, n (%)			*χ*^2^ = 0.06	.799
Married/cohabiting	98 (96.1)	105 (97.2)		
Other	4 (3.9)	3 (2.8)		
Education level, n (%)			*χ*^2^ = 0.91	.634
Secondary school or below	18 (17.6)	16 (14.8)		
College/University	63 (61.8)	66 (61.1)		
Postgraduate or above	21 (20.6)	26 (24.1)		
Employment status, n (%)			*χ*^2^ = 0.27	.603
Employed	71 (69.6)	79 (73.1)		
Unemployed/homemaker	31 (30.4)	29 (26.9)		
Monthly household income, n (%)			*χ*^2^ = 1.03	.597
< 5000 CNY	19 (18.6)	16 (14.8)		
5000–9999 CNY	44 (43.1)	46 (42.6)		
≥ 10,000 CNY	39 (38.2)	46 (42.6)		
Smoking exposure during pregnancy, n (%)	8 (7.8)	7 (6.5)	*χ*^2^ = 0.14	.708
Alcohol consumption during pregnancy, n (%)	5 (4.9)	4 (3.7)	*χ*^2^ = 0.18	.673
Family support score	7.86 ± 1.42	8.03 ± 1.37	*t* = 0.88	.379
Baseline obstetric risk factors, n (%)	24 (23.5)	22 (20.4)	*χ*^2^ = 0.30	.586
Advanced maternal age (≥ 35 yrs), n (%)	14 (13.7)	17 (15.7)	*χ*^2^ = 0.16	.692
Prepregnancy overweight/obesity, n (%)	29 (28.4)	26 (24.1)	*χ*^2^ = 0.50	.480
History of infertility treatment, n (%)	7 (6.9)	9 (8.3)	*χ*^2^ = 0.16	.693
History of gestational diabetes, n (%)	6 (5.9)	5 (4.6)	*χ*^2^ = 0.16	.688
History of hypertensive disorders of pregnancy, n (%)	4 (3.9)	3 (2.8)	*χ*^2^ = 0.20	.653
Family history of diabetes, n (%)	17 (16.7)	19 (17.6)	*χ*^2^ = 0.03	.870
Family history of hypertension, n (%)	21 (20.6)	23 (21.3)	*χ*^2^ = 0.02	.901
Baseline GAD-7 score	7.94 ± 3.26	7.63 ± 3.11	*t* = 0.71	.477
Baseline PHQ-9 score	7.21 ± 3.09	7.05 ± 2.96	*t* = 0.38	.703
Baseline health management compliance score	63.48 ± 8.75	64.31 ± 8.29	*t* = 0.71	.479

Data are presented as mean ± standard deviation or n (%).

BMI = body mass index, CNY = Chinese Yuan, GAD = generalized anxiety disorder, n = number of participants, PHQ = Patient Health Questionnaire.

### 3.2. Maternal psychological outcomes

Compared with the conventional care group, the intervention group showed significantly better maternal psychological outcomes. Specifically, the GAD-7 score was significantly lower in the intervention group than in the conventional care group (5.94 ± 2.87 vs 8.12 ± 3.41, *P* < .001). Similarly, the PHQ-9 score was significantly lower in the intervention group (5.71 ± 2.64 vs 7.46 ± 3.18, *P* < .001). In addition, the intervention group had significantly lower fear of childbirth scores (34.81 ± 7.93 vs 39.62 ± 8.75, *P* < .001) and perceived stress scores (17.92 ± 4.89 vs 20.73 ± 5.66, *P* < .001). The proportions of women with moderate-to-severe anxiety and moderate-to-severe depression were also significantly lower in the intervention group than in the conventional care group (13.0% vs 28.4%, *P* = .006; 10.2% vs 23.5%, *P* = .009, respectively) (Table 2).

**Table 2:** Comparison of maternal psychological outcomes between the 2 groups.

Outcome	Conventional care group (n = 102)	Intervention group (n = 108)	Statistic	*P* value
GAD-7 score	8.12 ± 3.41	5.94 ± 2.87	*t* = 5.03	< .001
PHQ-9 score	7.46 ± 3.18	5.71 ± 2.64	*t* = 4.35	< .001
Fear of childbirth score	39.62 ± 8.75	34.81 ± 7.93	*t* = 4.15	< .001
Perceived stress score	20.73 ± 5.66	17.92 ± 4.89	*t* = 3.86	< .001
Moderate-to-severe anxiety, n (%)	29 (28.4)	14 (13.0)	*χ*^2^ = 7.58	.006
Moderate-to-severe depression, n (%)	24 (23.5)	11 (10.2)	*χ*^2^ = 6.74	.009

Data are presented as mean ± standard deviation or n (%).

GAD = generalized anxiety disorder, n = number of participants, PHQ = Patient Health Questionnaire.

### 3.3. Pregnancy health management outcomes

The intervention group demonstrated significantly better performance in pregnancy health management indicators than the conventional care group. Adherence to scheduled antenatal visits was significantly higher in the intervention group than in the conventional care group (89.8% vs 76.5%, *P* = .009). The rate of appropriate gestational weight management was also higher in the intervention group (75.0% vs 59.8%, *P* = .019). In addition, compliance with health education recommendations was significantly better in the intervention group (85.2% vs 67.6%, *P* = .003). Exercise compliance and dietary management compliance were also significantly improved in the intervention group compared with the conventional care group (71.3% vs 51.0%, *P* = .002; 77.8% vs 55.9%, *P* = .001, respectively). Similarly, sleep management compliance and self-monitoring compliance were higher in the intervention group (73.1% vs 58.8%, *P* = .030; 75.9% vs 53.9%, *P* = .001, respectively). The mean number of antenatal visits was also significantly greater in the intervention group than in the conventional care group (9.6 ± 1.8 vs 8.7 ± 1.9, *P* = .001). Among women requiring blood glucose management, the intervention group showed a higher rate of satisfactory glycemic control than the conventional care group (79.5% vs 61.3%), although the difference did not reach statistical significance (*P* = .100).(Table 3)

**Table 3:** Comparison of pregnancy health management indicators between the 2 groups.

Indicator	Conventional care group (n = 102)	Intervention group (n = 108)	Statistic	*P* value
Adherence to scheduled antenatal visits, n (%)	78 (76.5)	97 (89.8)	*χ*^2^ = 6.77	.009
Appropriate gestational weight management, n (%)	61 (59.8)	81 (75.0)	*χ*^2^ = 5.50	.019
Compliance with health education, n (%)	69 (67.6)	92 (85.2)	*χ*^2^ = 9.10	.003
Satisfactory blood glucose control*, n (%)	19/31 (61.3)	31/39 (79.5)	*χ*^2^ = 2.71	.100
Exercise compliance, n (%)	52 (51.0)	77 (71.3)	*χ*^2^ = 9.24	.002
Dietary management compliance, n (%)	57 (55.9)	84 (77.8)	*χ*^2^ = 11.49	.001
Sleep management compliance, n (%)	60 (58.8)	79 (73.1)	*χ*^2^ = 4.74	.030
Self-monitoring compliance, n (%)	55 (53.9)	82 (75.9)	*χ*^2^ = 10.99	.001
Mean number of antenatal visits	8.7 ± 1.9	9.6 ± 1.8	*t* = 3.51	.001

n = number of participants.

* Among participants requiring blood glucose management.

### 3.4. Implementation-related and acceptability-related indicators

The intervention group also showed better implementation-related and acceptability-related indicators. The follow-up completion rate was significantly higher in the intervention group than in the conventional care group (94.4% vs 81.4%, *P* = .004). Attendance at nutritional counseling was significantly higher in the intervention group (88.0% vs 62.7%, *P* < .001). Likewise, attendance at psychological support sessions and stage-specific follow-up was significantly higher in the intervention group than in the conventional care group (82.4% vs 27.5%, *P* < .001; 90.7% vs 71.6%, *P* < .001, respectively). Management compliance was also significantly better in the intervention group (86.1% vs 66.7%, *P* = .001). In addition, the intervention group had a significantly higher satisfaction score than the conventional care group (91.46 ± 5.83 vs 84.27 ± 7.94, *P* < .001), and the proportion of women with high satisfaction was also markedly higher in the intervention group (70.4% vs 35.3%, *P* < .001). Conversely, the withdrawal or noncompletion rate was significantly lower in the intervention group (5.6% vs 18.6%, *P* = .004).(Table 4)

**Table 4:** Comparison of implementation-related and acceptability-related indicators.

Indicator	Conventional care group (n = 102)	Intervention group (n = 108)	Statistic	*P* value
Follow-up completion rate, n (%)	83 (81.4)	102 (94.4)	*χ*^2^ = 8.16	.004
Attendance at nutritional counseling, n (%)	64 (62.7)	95 (88.0)	*χ*^2^ = 18.31	< .001
Attendance at psychological support sessions, n (%)	28 (27.5)	89 (82.4)	*χ*^2^ = 63.71	< .001
Attendance at stage-specific follow-up, n (%)	73 (71.6)	98 (90.7)	*χ*^2^ = 12.25	< .001
Management compliance, n (%)	68 (66.7)	93 (86.1)	*χ*^2^ = 10.95	.001
Satisfaction score	84.27 ± 7.94	91.46 ± 5.83	*t* = 7.42	< .001
High satisfaction (≥ 90), n (%)	36 (35.3)	76 (70.4)	*χ*^2^ = 25.78	< .001
Withdrawal/noncompletion, n (%)	19 (18.6)	6 (5.6)	*χ*^2^ = 8.16	.004

Data are presented as mean ± standard deviation or n (%).

n = number of participants.

### 3.5. Maternal outcomes

With respect to maternal outcomes, several event rates were numerically lower in the intervention group than in the conventional care group, but none of these between-group differences reached statistical significance. The incidence of gestational diabetes mellitus was 23.1% in the intervention group and 30.4% in the conventional care group (*P* = .234). The incidence of hypertensive disorders of pregnancy was 7.4% in the intervention group and 11.8% in the conventional care group (*P* = .277). The cesarean section rate was 28.7% in the intervention group and 40.2% in the conventional care group (*P* = .078). Rates of preterm birth (5.6% vs 10.8%), postpartum hemorrhage (3.7% vs 6.9%), premature rupture of membranes (8.3% vs 14.7%), gestational anemia (13.9% vs 19.6%), and operative vaginal delivery (3.7% vs 5.9%) were also numerically lower in the intervention group, but these differences were not statistically significant (all *P* > .05). These numerical differences should therefore be interpreted descriptively and not as evidence of clinical effectiveness for maternal outcomes. (Table 5)

**Table 5:** Comparison of maternal outcomes between the 2 groups.

Outcome	Conventional care group (n = 102)	Intervention group (n = 108)	Statistic	*P* value
Gestational diabetes mellitus, n (%)	31 (30.4)	25 (23.1)	*χ*^2^ = 1.42	.234
Hypertensive disorders of pregnancy, n (%)	12 (11.8)	8 (7.4)	*χ*^2^ = 1.18	.277
Cesarean section, n (%)	41 (40.2)	31 (28.7)	*χ*^2^ = 3.10	.078
Preterm birth, n (%)	11 (10.8)	6 (5.6)	*χ*^2^ = 1.89	.169
Postpartum hemorrhage, n (%)	7 (6.9)	4 (3.7)	*χ*^2^ = 1.05	.305
Premature rupture of membranes, n (%)	15 (14.7)	9 (8.3)	*χ*^2^ = 2.09	.148
Gestational anemia, n (%)	20 (19.6)	15 (13.9)	*χ*^2^ = 1.23	.267
Operative vaginal delivery, n (%)	6 (5.9)	4 (3.7)	*χ*^2^ = 0.54	.463

Data are presented as n (%).

n = number of participants.

### 3.6. Neonatal outcomes

No statistically significant differences were observed in neonatal outcomes. The mean birth weight was 3326 ± 401 g in the intervention group and 3278 ± 436 g in the conventional care group (*P* = .405). The rates of macrosomia and low birth weight were 5.6% vs 8.8% and 4.6% vs 7.8%, respectively, with no statistically significant between-group differences. NICU admission occurred in 4.6% of the intervention group and 9.8% of the conventional care group (*P* = .147). The 1-min and 5-min Apgar scores were comparable between groups (*P* = .163 and *P* = .373, respectively). Neonatal jaundice (9.3% vs 12.7%) and fetal distress (4.6% vs 8.8%) also showed numerical differences, but these were not statistically significant. Accordingly, these findings are descriptive and should not be interpreted as demonstrating a neonatal benefit of the intervention. (Table 6)

**Table 6:** Comparison of neonatal outcomes between the 2 groups.

Outcome	Conventional care group (n = 102)	Intervention group (n = 108)	Statistic	*P* value
Birth weight (g)	3278 ± 436	3326 ± 401	*t* = 0.83	.405
Macrosomia, n (%)	9 (8.8)	6 (5.6)	*χ*^2^ = 0.83	.362
Low birth weight, n (%)	8 (7.8)	5 (4.6)	*χ*^2^ = 0.95	.330
NICU admission, n (%)	10 (9.8)	5 (4.6)	*χ*^2^ = 2.10	.147
1-min Apgar score	9.61 ± 0.62	9.72 ± 0.51	*t* = 1.40	.163
5-min Apgar score	9.93 ± 0.29	9.96 ± 0.20	*t* = 0.89	.373
Neonatal jaundice, n (%)	13 (12.7)	10 (9.3)	*χ*^2^ = 0.65	.421
Fetal distress, n (%)	9 (8.8)	5 (4.6)	*χ*^2^ = 1.45	.229

Data are presented as mean ± standard deviation or n (%).

n = number of participants, NICU = neonatal intensive care unit.

### 3.7. Multivariable analysis

After adjustment for potential confounding factors, including age, prepregnancy BMI, parity, and baseline obstetric risk factors, the intervention model remained independently associated with better antenatal care adherence (OR = 2.84, 95% CI: 1.29–6.24, *P* = .009) and improved gestational weight management (OR = 1.96, 95% CI: 1.09–3.54, *P* = .025). In the linear regression analysis, the intervention model was also significantly associated with lower GAD-7 scores (*β* = −1.87, 95% CI: −2.73–−1.01, *P* < .001). Prepregnancy BMI was negatively associated with appropriate gestational weight management (OR = 0.92, 95% CI: 0.86–0.99, *P* = .032), while baseline obstetric risk factors were positively associated with higher GAD-7 scores (*β* = 0.91, 95% CI: 0.12–1.70, *P* = .024). (Table 7)

**Table 7:** Multivariable analysis of factors associated with key outcomes.

Outcome	Variable	OR/*β*	95% CI	*P* value
Adherence to antenatal visits	Intervention model	2.84	1.29–6.24	.009
	Age	1.03	0.96–1.11	.389
	Prepregnancy BMI	0.97	0.89–1.05	.452
	Primipara	1.22	0.61–2.45	.570
Gestational weight management	Intervention model	1.96	1.09–3.54	.025
	Age	1.01	0.95–1.08	.692
	Prepregnancy BMI	0.92	0.86–0.99	.032
	Obstetric risk factors	0.79	0.38–1.64	.524
GAD-7 score	Intervention model	−1.87	−2.73–−1.01	< .001
	Age	−0.05	−0.14–0.03	.227
	Prepregnancy BMI	0.08	−0.03–0.19	.149
	Obstetric risk factors	0.91	0.12–1.70	.024

β: regression coefficient.

BMI = body mass index, CI = confidence interval, GAD = generalized anxiety disorder, OR = odds ratio.

## 4. Discussion

This retrospective comparative study evaluated the clinical application of a full-course precision MDT nursing studio model integrating REBT for standardized health management during pregnancy. Baseline demographic, obstetric, psychological, and health management characteristics were generally comparable between the 2 groups, providing a reasonable basis for subsequent between-group comparisons. Within this context, the main differences were observed in maternal psychological indicators, pregnancy health management indicators, and implementation-related measures. Specifically, women in the intervention group had lower GAD-7 and PHQ-9 scores, lower fear of childbirth scores, lower perceived stress scores, and lower proportions of moderate-to-severe anxiety and depression. These findings are clinically meaningful because emotional distress during pregnancy is common, often co-occurs with other adjustment difficulties, and may affect treatment adherence, self-management behavior, and the broader pregnancy experience. Recent evidence has shown that cognitive-behavioral and other structured psychological interventions can reduce perinatal depressive and anxiety symptoms, including in antenatal populations, and may exert effects across different delivery formats and levels of intensity.^[11]^ In this context, integrating REBT into pregnancy care appears theoretically plausible, as the approach directly targets maladaptive cognitions, catastrophic interpretations, and rigid beliefs that may arise around fetal health, bodily symptoms, childbirth, and maternal role transition. The present findings are therefore broadly consistent with the current literature on perinatal psychological interventions and cognitive-behavioral approaches.^[12–15]^

Another important finding was the pattern observed in pregnancy health management indicators. Compared with the conventional care group, the intervention group had higher adherence to scheduled antenatal visits, a higher rate of appropriate gestational weight management, and higher compliance with health education, exercise, dietary management, sleep management, and self-monitoring. The mean number of antenatal visits was also higher in the intervention group. These results suggest that the effect of the intervention was not confined to emotional support but extended to behavioral participation in routine pregnancy management. This interpretation is in line with the broader perinatal care literature showing that nonpharmacological and educational interventions may influence both psychological outcomes and aspects of care engagement.^[16–18]^ Recent evidence suggests that interventions such as antenatal education, nonpharmacological prenatal support, and primary care-based nondrug approaches can reduce fear of childbirth and anxiety while strengthening confidence and participation in care.^[19]^ Likewise, reviews of midwifery continuity and continuity-based maternity models indicate that ongoing relationships with providers and structured follow-up may support maternal mental health and selected care processes.^[20,21]^ Although the difference in satisfactory blood glucose control did not reach statistical significance in the present study, the numerical direction was similar to that of the other management-related variables. This may reflect the smaller subgroup size and the multifactorial determinants of glycemic control during pregnancy, including baseline metabolic risk, treatment intensity, and adherence to dietary and activity recommendations. Overall, the pattern across these indicators supports the view that whole-course, coordinated antenatal management may influence multiple dimensions of pregnancy-related self-care.^[22]^

The implementation-related and acceptability-related findings also warrant attention. The intervention group had higher follow-up completion, higher attendance at nutritional counseling, higher attendance at psychological support sessions, higher attendance at stage-specific follow-up, higher management compliance, and higher satisfaction scores, while withdrawal or noncompletion was less frequent. These observations are relevant because the practical value of a clinical nursing model depends not only on measurable outcomes, but also on whether the intervention can be integrated into routine care and whether women are willing and able to remain engaged throughout pregnancy. Contemporary research on integrated perinatal mental health care emphasizes the importance of coordinated service models, clear referral pathways, culturally and psychologically safe care, and implementation strategies that reduce fragmentation across obstetric and mental health services.^[23]^ Evidence syntheses have also identified multiple barriers and facilitators influencing the implementation of perinatal mental health care, including training, trusting relationships, accessibility, stigma, and organizational support.^[24]^ In addition, trauma-informed guidance has highlighted the need for care models that recognize pregnancy-related trauma and emotional vulnerability while maintaining continuity and responsiveness in service delivery.^[25]^ The present intervention, which was delivered through a nursing studio framework and embedded within the existing antenatal pathway, may therefore reflect several features that are increasingly emphasized in the integrated care literature.^[22–24]^

For maternal and neonatal outcomes, several variables showed numerical differences between groups, including gestational diabetes mellitus, hypertensive disorders of pregnancy, cesarean section, preterm birth, postpartum hemorrhage, premature rupture of membranes, gestational anemia, NICU admission, macrosomia, low birth weight, neonatal jaundice, and fetal distress. However, most of these differences did not reach statistical significance and should not be interpreted as evidence that the intervention improves maternal or neonatal clinical endpoints. Compared with psychological indicators and health management measures, maternal and neonatal endpoints are influenced by a broader range of biological, clinical, and obstetric factors, and a moderate sample size in a retrospective single-center study may be insufficient to detect differences in relatively infrequent outcomes. Existing evidence on digital or remote mental health support and on broader perinatal intervention strategies similarly suggests that measurable benefits are often more consistently observed for proximal outcomes such as symptom scores, self-efficacy, help-seeking, or participation in care than for harder obstetric endpoints.^[25]^ The multivariable analyses in the present study showed associations with antenatal care adherence, gestational weight management, and GAD-7 score after adjustment for selected maternal characteristics; these observational associations do not establish causality. Several limitations should be acknowledged. First, because of the retrospective, non-randomized design, causal relationships cannot be established, and selection bias cannot be excluded. The available analytic records identified the care model received but did not reliably capture the operational reason for allocation to one care model or the other. Second, the study was conducted at a single tertiary maternity center, which may limit generalizability. Third, some variables were derived from routine clinical records and follow-up documentation, and incomplete recording cannot be entirely excluded. The follow-up timing of psychological assessments was not linked to a uniform gestational-week variable in the analytic dataset, so timing variability may have affected between-group comparability. In addition, several management, fear of childbirth, family support, and satisfaction indicators were based on routine institutional tools rather than uniformly validated research instruments; variation in how these measures were recorded or classified may reduce reproducibility across settings. Residual confounding may also remain despite multivariable adjustment. Finally, the intervention was multicomponent in nature, and the present study could not disentangle the relative contribution of the MDT framework, the REBT component, and the repeated follow-up process. Future studies would benefit from prospective multicenter designs, larger samples, prespecified and synchronized assessment time points, validated measurement instruments, and more detailed longitudinal assessment of psychological, behavioral, and obstetric trajectories across pregnancy.^[22,25,26]^

## 5. Conclusion

In this retrospective comparative study, the full-course precision MDT nursing studio model integrating REBT was associated with better maternal psychological status, improved antenatal care adherence, more favorable pregnancy health management performance, and higher implementation-related indicators during standardized antenatal care. Several maternal and neonatal outcomes showed numerical between-group differences, but most were not statistically significant; therefore, no conclusion regarding clinical effectiveness for these endpoints can be drawn from the present data. Given the observational design, possible selection and measurement variability, and the multicomponent nature of the intervention, these findings should be interpreted cautiously. Larger prospective multicenter studies are needed to confirm these associations and further define the role of this integrated care model in pregnancy health management.

## Author contributions

**Conceptualization:** Li Zhang, Hongying Tao, Lingna Zhu, Shoufu Liu, Yanhui Yang.

**Data curation:** Li Zhang, Hongying Tao, Lingna Zhu, Shoufu Liu, Yanhui Yang.

**Formal analysis:** Li Zhang, Hongying Tao, Lingna Zhu, Shoufu Liu, Yanhui Yang.

**Funding acquisition:** Yanhui Yang.

**Investigation:** Yanhui Yang.

**Writing** – **original draft:** Yanhui Yang.

**Writing** – **review & editing:** Yanhui Yang.
